# Habitat-specific variation in gut microbial communities and pathogen prevalence in bumblebee queens (*Bombus terrestris*)

**DOI:** 10.1371/journal.pone.0204612

**Published:** 2018-10-25

**Authors:** L. Bosmans, M. I. Pozo, C. Verreth, S. Crauwels, L. Wilberts, I. S. Sobhy, F. Wäckers, H. Jacquemyn, B. Lievens

**Affiliations:** 1 Laboratory for Process Microbial Ecology and Bioinspirational Management (PME&BIM), Department of Microbial and Molecular Systems, KU Leuven, Campus De Nayer, Sint-Katelijne-Waver, Belgium; 2 Plant Conservation and Population Biology, Biology Department, KU Leuven, Heverlee, Belgium; 3 Department of Plant Protection, Faculty of Agriculture, Suez Canal University, Ismailia, Egypt; 4 Lancaster Environment Centre, Lancaster University, Lancaster, United Kingdom; 5 Biobest Group, Westerlo, Belgium; University of California San Diego, UNITED STATES

## Abstract

Gut microbial communities are critical for the health of many insect species. However, little is known about how gut microbial communities respond to anthropogenic changes and how such changes affect host-pathogen interactions. In this study, we used deep sequencing to investigate and compare the composition of gut microbial communities within the midgut and ileum (both bacteria and fungi) in *Bombus terrestris* queens collected from natural (forest) and urbanized habitats. Additionally, we investigated whether the variation in gut microbial communities under each habitat affected the prevalence of two important bumblebee pathogens that have recently been associated with *Bombus* declines (*Crithidia bombi* and *Nosema bombi*). Microbial community composition differed strongly among habitat types, both for fungi and bacteria. Fungi were almost exclusively associated with bumblebee queens from the forest habitats, and were not commonly detected in bumblebee queens from the urban sites. Further, gut bacterial communities of urban *B*. *terrestris* specimens were strongly dominated by bee-specific core bacteria like *Snodgrassella* (Betaproteobacteria) and *Gilliamella* (Gammaproteobacteria), whereas specimens from the forest sites contained a huge fraction of environmental bacteria. Pathogen infection was very low in urban populations and infection by *Nosema* was only observed in specimens collected from forest habitats. No significant relationship was found between pathogen prevalence and microbial gut diversity. However, there was a significant and negative relationship between prevalence of *Nosema* and relative abundance of the core resident *Snodgrassella*, supporting its role in pathogen defense. Overall, our results indicate that land-use change may lead to different microbial gut communities in bumblebees, which may have implications for bumblebee health, survival and overall fitness.

## Introduction

Insects represent one of the most species-rich animal groups on Earth [1, 2] and play an important role in ecosystem functioning [3]. Insects provide numerous important ecosystem services such as pollination, crop protection and detrivory, nutrient cycling, and providing a food source for higher trophic levels [4, 5]. Recent studies have suggested that insects are drastically declining worldwide [6], particularly pollinators [7]. Habitat loss and fragmentation, decline in food resources and nest availability, and increased use of pesticides, as well as climate change have been proposed as the most important factors leading to insect decline [8]. Recently, increased prevalence of diseases and parasites has been suggested to contribute to pollinator decline as well [9]. However, there is increasing consensus that there is likely no single factor that can explain this severe decline, but rather a complex interaction of many factors that act together [8].

While effects of anthropogenic disturbances on insect diversity have been extensively studied [10, 11], little is known about the effect of land-use change on the microbes that live in close association with insects and how anthropogenic changes affect pathogen infection in insects. It is known that diverse microbial communities inhabit insect guts (but exceptions exist, see [12]) and that they can provide critical functions for their hosts, such as nutrient acquisition, food digestion, regulation of immune responses, insecticide resistance and defense against pathogens and parasites [13–17]. They can also influence host development, behaviour and reproduction [14, 18, 19]. Disruption or changes of the gut microbiome may therefore have important consequences for the host, often resulting in adverse health and fitness effects [20]. The exact species composition of the gut microbiome is influenced by many factors, including diet, host genetics, immune responses, stress, exposure to antimicrobials, interactions between members of the gut microbial community, and other factors such as flower availability, local environmental conditions and the pool of environmental bacteria that may invade and colonize the insect gut [19, 21–23]. It can therefore be hypothesized that land-use conversion affects the gut microbial community composition, and therefore also defense against pathogens and overall health [21].

The overall objective of this study was to determine whether gut microbial communities of wild bumblebee queens are influenced by urbanization, and whether potential differences in community composition translate in different pathogen prevalence. Previous research has shown that bumblebees harbour a specialized, species-poor gut microbial community, which is dominated by a limited set of bee-specific core bacteria, including *Gilliamella* (Gamma-1 phylotype; Gammaproteobacteria; *Orbaceae*), *Snodgrassella* (Beta; Gammaproteobacteria; *Neisseriaceae*), *Lactobacillus* (Firm-4/Lacto-2 and Firm-5/Lacto-1; Firmicutes; *Lactobacillaceae*) and bifidobacteria (Actinobacteria) [24–27]. Cariveau *et al*. [26] found that anthropogenic change only marginally affected the gut microbial communities of *Bombus* workers. However, in their study only the effect of agricultural activities was investigated, while effects of other anthropogenic disturbances such as urbanization remain to be studied, as well as effects on queens. We first examined and compared the composition of gut microbial communities within the midgut and ileum (both bacteria and fungi) in *Bombus terrestris* queens that were collected from natural (forest) and urbanized habitats. Next, we investigated whether habitat type and variation in gut microbial community composition affected the occurrence of two important bumblebee pathogens, *Crithidia bombi* (Kinetoplastida; *Trypanosomatidae*) and *Nosema bombi* (Microsporidia; *Nosematidae*). Together with a few other pathogens like *Apicystis bombi* (Neogregarinorida; *Ophryocystidae*), these two species are believed to have contributed to the observed declines of *Bombus* populations [28, 29]. Previous research has shown that infection by these pathogens may be related to the gut microbial community composition, although relationships were not always significant [17, 24, 26, 30].

## Materials and methods

### Study species and collection of specimens

Experiments were performed using queens of *Bombus terrestris* (L.) (Hymenoptera: *Apidae*). *Bombus terrestris* is one of the most numerous bumblebee species in Europe and known to be an important pollinator of several wild plants and crops [31]. Specimens were collected in early spring (March/April) within 7 days when the first queens that emerged from hibernation were observed in the field. No permission was required to perform the sampling; the sampling dit not involve endangered or protected species. Indiviuals were sampled from two natural (forest) locations (further referred to as site “S1-F” (Heverlee) and “S2-F” (Viroin)) and three urbanized areas (“S3-U” (Sint-Katelijne-Waver), “S4-U” (Leuven) and “S5-U” (Leuven)) in Belgium (S1 Table). Sites were separated by a mean of 46 km (3.0–160 km). At the time of sampling, very few plant species such as *Narcissus pseudonarcissus* and *Helleborus foetidus* were flowering at the forest locations while *Erica carnea* and a few other species were flowering in gardens of the urban areas that were sampled. In total, ten individual queens were collected per location, except for forest location S1-F where eight queens were collected. Specimens were individually put in plastic 50 ml vials and transported in a cooling box to the laboratory to avoid any mortality incidences by stress.

### Gut dissection, DNA extraction, PCR amplification and Illumina MiSeq analysis

Following rinsing with 70% ethanol, each specimen (alive, fresh specimens) was pinned to a polyacrylamide gel plate and immersed in sterile Ringer’s solution. Subsequently, the intestines were pulled out and the midgut and hindgut excluding rectum (i.e. the ileum region) were collected into a vial with 1 ml glycerol (40%) and homogenized by using zirconia beads and a Fast-Prep24 Instrument (MP Biomedicals, USA). The dissections were conducted under a binocular microscope (M420 Wild Makroskop, Switzerland). After every dissection, Ringer’s solution was replaced and the gel plate was cleaned and desinfected with 70% ethanol. Next, guts were crushed in 170 μl lysozyme solution (100 mg/ml) and DNA was extracted according to Meeus *et al*. [18]. A negative control was included during extraction in which the lysozyme solution without gut material was used as starting material. DNA samples were then subjected to PCR amplification and Illumina MiSeq sequencing. Again a negative control was included (PCR amplification control), this time by replacing template DNA with sterile water. Results obtained for both types of negative controls were satisfactory, confirming that the experimental conditions were met to achieve robust data. DNA samples were amplified using sample-specific barcode-labeled versions of the primer sets 515F / 806R [32] and ITS86F / ITS4 [33], generating amplicons covering the V4 region of the bacterial 16S rRNA gene and the fungal internal transcribed spacer-2 (ITS-2) region, respectively (dual-index sequencing strategy [34]; S2 Table). Amplification was performed in a volume of 40 μl containing 1x Titanium Taq PCR buffer, 150 μM of each dNTP, 0.5 μM of each primer, 1x Titanium Taq DNA polymerase (Clontech, Saint-Germain-en-Laye, France) and 2 μl DNA (5 ng μl^-1^), using the following thermal profile: initial denaturation at 94°C for 2 min, followed by 30 cycles of denaturation at 94°C for 45 s, annealing at 59°C for 45 s and elongation at 72°C for 45 s, and terminated by a final elongation at 72°C for 10 min. PCR amplicons were then purified using Agencourt AMPure XP magnetic beads (Beckman Coulter Genomics GmbH, South Plainfield, UK) according to the manufacturer’s guidelines. Following quantification of the purified products using a Qubit High Sensitivity Fluorometer kit (Invitrogen, Carlsbad, CA, USA) amplicons were combined at equimolar concentrations into two amplicon libraries (one for bacteria and one for fungi). Subsequently, the libraries were subjected to an ethanol precipitation and loaded on an agarose gel. Next, the target bands (*c*. 250 bp for bacteria, *c*. 400 bp for fungi) were excised and the DNA was purified and measured again. Amplicon mixtures were then combined, diluted to 2 nM and sequenced using an Illumina MiSeq sequencer with v2 500 cycle reagent kit (Illumina, San Diego, CA, USA).

Sequences were received as a de-multiplexed FASTQ file. Paired-end reads were merged with a maximum of five mismatches using USEARCH (v10.0.240) to form consensus sequences [35] and truncated at the 250th base. Shorter reads or reads with a total expected error threshold above 0.05 (for 16S rRNA gene sequences) or above 0.10 (for ITS) were discarded using VSEARCH (v2.4.0) [36]. Next, in order to remove potential contaminant sequences, sequences were classified with the Mothur (v1.36.1) implementation of the Silva database (v1.23) (for 16S rRNA gene sequences) and the RDP Warcup fungal ITS training set (v2) [37] (for ITS sequences) using the “classify.seqs” command. Subsequently, 16S rRNA gene sequences identified as “mitochondria,” “chloroplast,” “archaea,” “eukaryota,” or “unknown” were removed using the “remove.lineage” command. For ITS sequences, only sequences that could be identified to the fungal family level with a bootstrap confidence value of 100% were retained using the “get.lineage” command. Next, the number of sequences was rarefied to that of the sample with the lowest number of reads (*c*. 23,350 for bacteria and 2,000 for fungi), and sequences were grouped into operational taxonomic units (OTUs) based on a 3% sequence dissimilarity cut-off using the UPARSE greedy algorithm in USEARCH, during which chimeric sequences were also removed [35], as were global singletons (i.e. OTUs with only 1 sequence in the entire data set). The taxonomic origin of each OTU was determined with the SINTAX algorithm implemented in USEARCH [38], based on the Silva v1.23 database [39] and the RDP Warcup fungal ITS training set (v2) [37] for bacteria and fungi, respectively. In general, taxonomic assignments can be considered reliable when bootstrap confidence values exceed 0.80. Furthermore, BLAST searches were performed against type materials in GenBank verifying the identity of the most important OTUs. Additionally, for core bacteria, identifications were refined up to phylotypes by available information in the literature and GenBank [26, 27]. Raw sequence data were deposited in the Sequence Read Archive under BioProject accession PRJNA445658. Representative OTU sequence data (selected by UPARSE) were deposited in Genbank under the accession MH815148 to MH816704.

### qPCR to estimate bacterial and fungal abundance

Quantitative real-time PCR (qPCR) was used to estimate total bacterial and fungal abundance in the gut samples, as well as the abundance of the core bacteria *Gilliamella* and *Snodgrasella*. Specifically, the universal bacterial primers 519F and 907R were used to amplify total copies of the16S rRNA gene. Additionally, Beta-1009-qtF and Beta-1115-qtR [25] and LWI1_1 (5’-TAC GGA GGG TGC GAG CGT T-3’) and Gamma1-648-qtR [25] were used to amplify the 16S rRNA genes of *Snodgrassella* and *Gilliamella*, respectively. Further, ITS86F and ITS4 were used to amplify total fungal ITS-2 copies. qPCR amplifications were performed in MicroAmp Fast 8-Tube Strips (Life Technologies, Carlsbad, CA, USA) using a StepOnePlus real-time PCR system (Applied Biosystems, Carlsbad, CA, USA), and each reaction was performed in duplicate. Each reaction contained 1.0 μl DNA (5 ng), 10.0 μl of the iTaq Universal SYBRGreen supermix (Bio-Rad, Hercules, CA, USA), 0.2 μl of each primer (20 μM) and 8.6 μl sterile water. Thermal cycling conditions consisted of 2 min at 95°C, followed by 40 amplification cycles of 15 s at 95°C, 30 s at 59°C (519F/907R; ITS86F/ITS4) or 55°C (Beta-1009-qtF/Beta-1115-qtR; LWI1_1/Gamma1-648-qtR) and 30 s at 60°C. Fluorescence (520 nm) was detected at the end of the elongation phase for each cycle. To evaluate amplification specificity, a melting curve analysis was performed at the end of each PCR run. In each analysis, a positive and negative control (template DNA replaced by sterile water) was included. Quantification was based on standard curves from amplification of cloned target sequences in a TOPO-TA vector (Invitrogen).

### Pathogen assessment

In order to evaluate the prevalence of *N*. *bombi* and *C*. *bombi*, qPCR was performed using the primers developed by Huang *et al*. [40]. Amplifications were performed as described above, with the exception of the annealing temperature which was 64.5°C. Each reaction was performed in duplicate, and in each analysis, a positive (pathogen DNA) and negative control (template DNA replaced by sterile water) was included. Additionally, six gut samples from commercially reared *B*. *terrestris* queens (Biobest Group, Westerlo, Belgium), that were known to be free of pathogens (screened by microscopy), were included as a negative control, and were also found to be free of pathogens using the qPCR (S1 Table). As previous analyses had shown that the primers may yield aspecific products (data not shown), all amplicons obtained were sequenced to confirm the identity of the pathogens. A sample was considered positive when both the C_T_ value was below that of the negative control (sterile water) and amplicon sequencing confirmed pathogen identity.

### Statistical analyses

#### Community composition

Rarefaction curves were constructed for the bacterial and fungal communities using the Vegan package (v2.4–6) for R (R Development Core Team, 2013). Additionally, for each sample, OTU richness (*S*), the Chao1 estimator, Shannon-Wiener diversity (*H*) and Pielou’s evenness (*J* = *H*/ln(*S*)) were calculated using USEARCH (v10.0.240) [35] and compared using a mixed model analysis of variance with habitat type (forest or urban environment) as fixed factor and population as random factor. To this end, Shannon diversity was exponentially transformed (exp(*H*)) to obtain a linear variable [41]. Non-metric multidimensional scaling (NMDS) was used to visualize the level of similarity in community composition between the different samples based on Bray-Curtis similarities (relative abundance data). Further, permutational multivariate analysis of variance (PERMANOVA) using the adonis function in the vegan package was performed to test for significant differences in microbial gut community composition between the different locations.

#### Disease prevalence

To test the hypothesis that disease prevalence was associated with habitat type, we used a logistic regression analysis, with habitat type as fixed factor and presence/absence of the disease as dependent variable. Analyses were performed for each disease separately. Secondly, we investigated whether disease occurrence was related to gut microbial diversity. Finally, we tested the hypothesis that pathogen occurence was related to the abundance of core bacteria in the gut that were previously suggested to protect the bees against gut pathogens and parasites [24, 26, 30]. We used logistic regression analyses with the relative abundance of the two main core gut bacteria (*Snodgrassella* and *Gilliamella*) as independent variable and the presence/absence of *N*. *bombi* and *C*. *bombi* as dependent variable. Analyses were performed for each pathogen separately.

## Results

### Bacterial community composition

**Fig 1:**
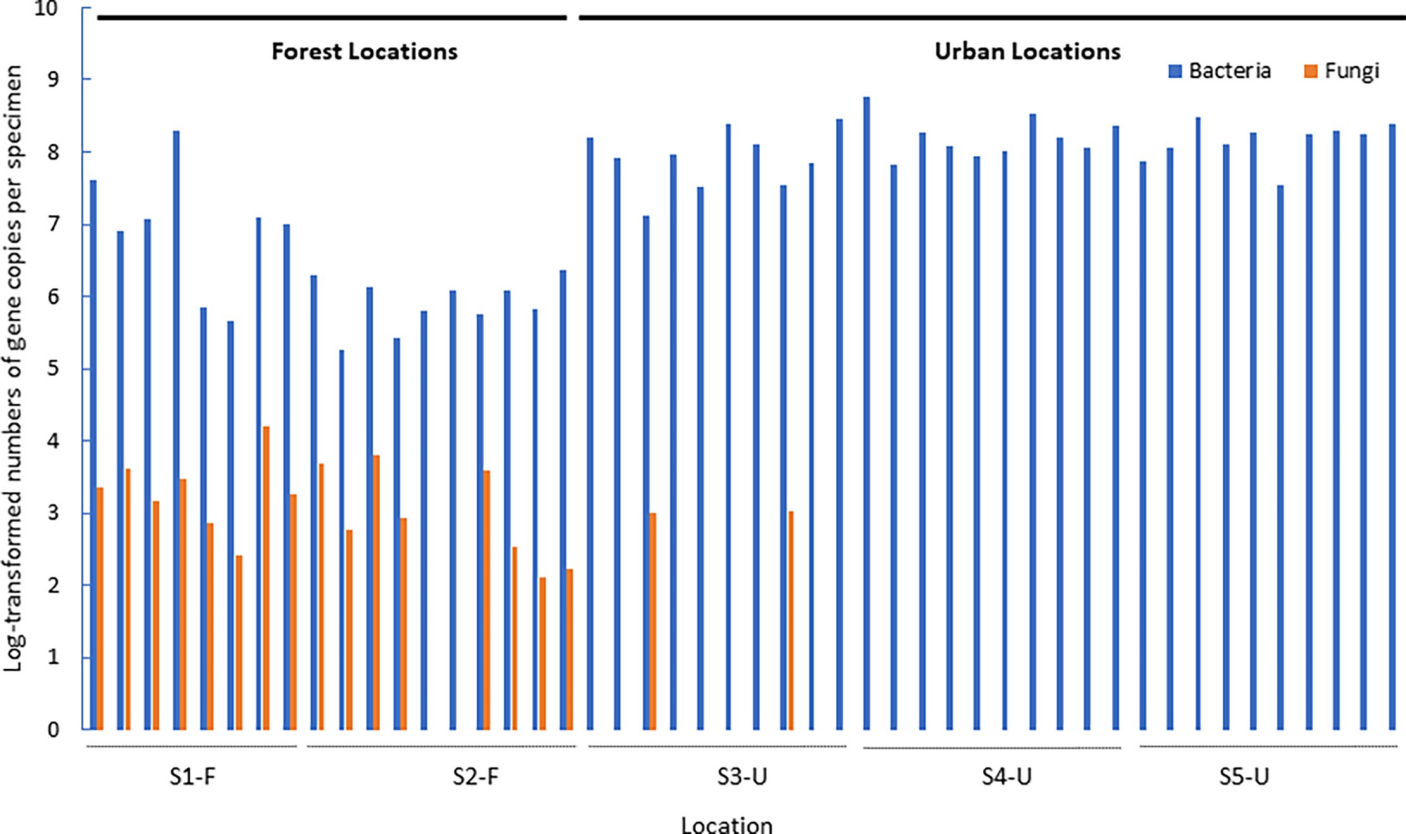
Means (*n* = 2) of log-transformed numbers of bacterial 16S rRNA gene and fungal ITS-2 copies in the midgut and ileum region per specimen. Bumblebee queens (*Bombus terrestris*) were collected from five different locations, representing two habitat types, including forest (S1-F and S2-F) and urbanized habitats (S3-U, S4-U and S5-U). No bars represent concentrations below the limit of detection (100 copies).

Analysis of the gut microbiota revealed a total of 1,450 bacterial OTUs belonging to diverse phyla (among which the most abundant were Acidobacteria, Bacteroidetes, Firmicutes and Proteobacteria) (S3 Table and S1 Fig). Based on Chao1, the mean sampling coverage was 86.8% (S1 Table), suggesting that the most abundant bacterial community members were covered, as can also be observed from the rarefaction curves that approached saturation (S2 Fig). Observed OTU richness varied between 14 and 589 bacterial OTUs (mean of 239 OTUs) per bumblebee in the forest locations, while sampled individuals from the urbanized locations contained between 4 and 153 bacterial OTUs (mean of 56 OTUs) (S1 Table). Furthermore, gut microbial communities were more evenly distributed (*F* = 1.87, *P* = 0.029) for bumblebee queens from the forest locations (Table 1; S1 Table). Results from the qPCR analysis indicated that log transformed total bacterial 16S rRNA gene copy numbers were signifcantly higher for the urban specimens (*F* = 1.63, *P* = 0.018). Gene copy numbers in the forest populations were 1 to 2 log orders lower compared to the urban populations (Fig 1).

**Table 1:** Mean diversity measures (± standard error of the mean) of the gut microbiome (midgut and ileum) of the bumblebee queens (*Bombus terrestris*) investigated in this study^a^.

	Sampling site	Habitat type	n^b^	Observed OTU richness (*S*)	Chao1	Coverage^c^ (%)	Shannon-Wiener (*H*)	Transformed S-W (exp(*H*))	Evenness (*J*)
Bacteria	S1-F	Natural area	8 of 8	241 ± 176 ^A^	242.8 ± 175.5 ^A^	98.5 ± 2.9	2.32 ± 1.58	25.03 ± 30.42 ^A^	0.42 ± 0.24 ^A^
	S2-F	Natural area	10 of 10	238 ± 49 ^A^	239.3 ± 48.8 ^A^	99.3 ± 1.3	2.88 ± 1.71	48.90 ± 53.87 ^A^	0.52 ± 0.31 ^A^
	S3-U	Urbanized area	10 of 10	66 ± 33 ^B^	90.6 ± 45.9 ^B^	79.0 ± 24.9	1.79 ± 0.77	7.53 ± 4.69 ^B^	0.43 ± 0.15 ^A^
	S4-U	Urbanized area	10 of 10	48 ± 45 ^B^	57.8 ± 44.1 ^B^	80.5 ± 23.0	0.88 ± 0.26	2.48 ± 0.65 ^B^	0.27 ± 0.05 ^B^
	S5-U	Urbanized area	10 of 10	53 ± 40 ^B^	62.8 ± 35.6 ^B^	79.0 ± 19.8	1.03 ± 0.23	2.86 ± 0.65 ^B^	0.28 ± 0.0 ^B^
Fungi	S1-F	Natural area	8 of 8	15 ± 9 ^A^	15.9 ± 10.1 ^A^	98.1 ± 3.7	1.40 ± 0.60	1.40 ± 0.60 ^A^	0.39 ± 0.17 ^A^
	S2-F	Natural area	10 of 10	31 ± 4 ^B^	33.9 ± 5.3 ^B^	93.1 ± 8.6	1.83 ± 0.74	1.83 ± 0.74 ^B^	0.52 ± 0.21 ^A^
	S3-U	Urbanized area	2 of 10	22 ± 6 ^B^	25.9 ± 8.9 ^C^	87.9 ± 16.3	1.17 ± 0.64	1.17 ± 0.64 ^C^	0.45 ± 0.04 ^A^
	S4-U	Urbanized area	0 of 10	-	-	-	-	-	-
	S5-U	Urbanized area	0 of 10	-	-	-	-	-	-

a Different letters indicate significant differences (mixed model analysis; P < 0.05). Data for bacteria and fungi were analyzed separately.

b Number of individuals included in the analysis.

c Coverage = (Observed OTU richness/Chao1)*100%.

**Fig 2:**
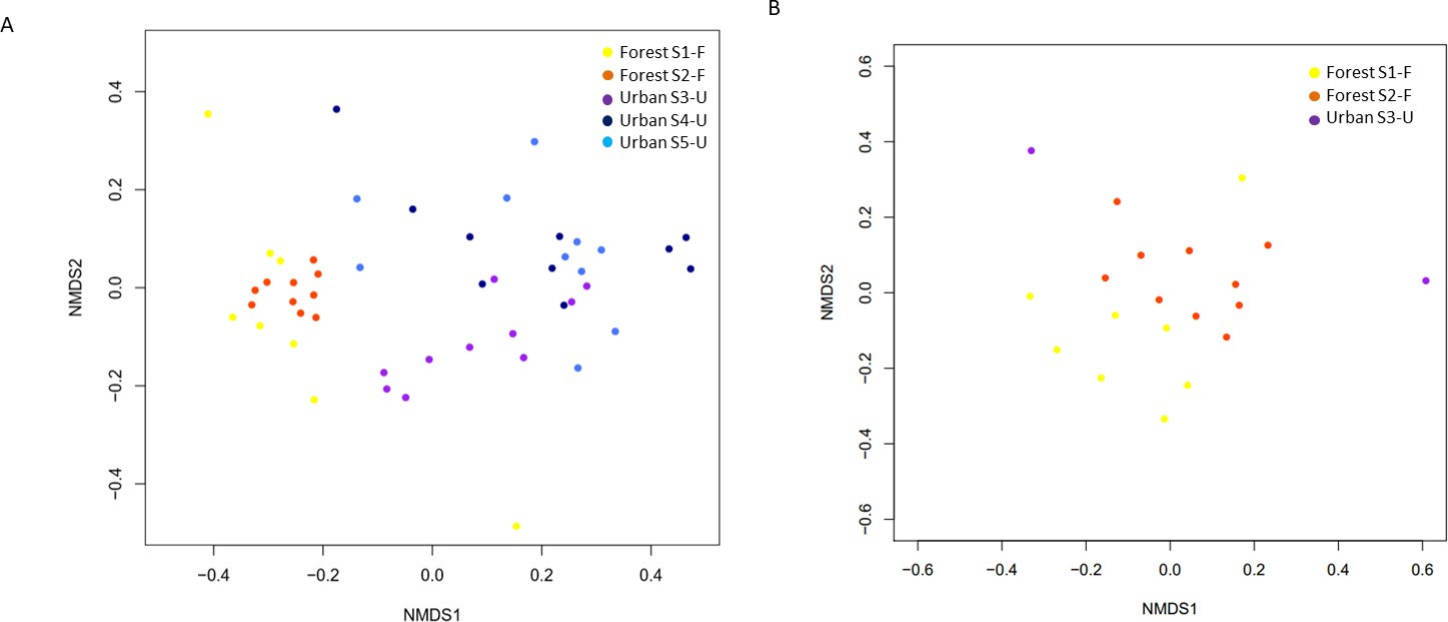
**Non-metric multidimensional scaling (NMDS) based on Bray-Curtis dissimilarities of the gut bacterial (A) (stress value = 0.16) and fungal community composition (B) (stress value = 0.21) of *Bombus terrestris* queens from five different locations**. Sampled locations represent two habitat types, including forest (S1-F (yellow) and S2-F (orange)) and urbanized habitats (S3-U (purple), S4-U (dark blue) and S5-U (light blue)). The distance between different points on the plot reflects their similarity level: the more similar the communities, the smaller the distance between the points.

**Fig 3:**
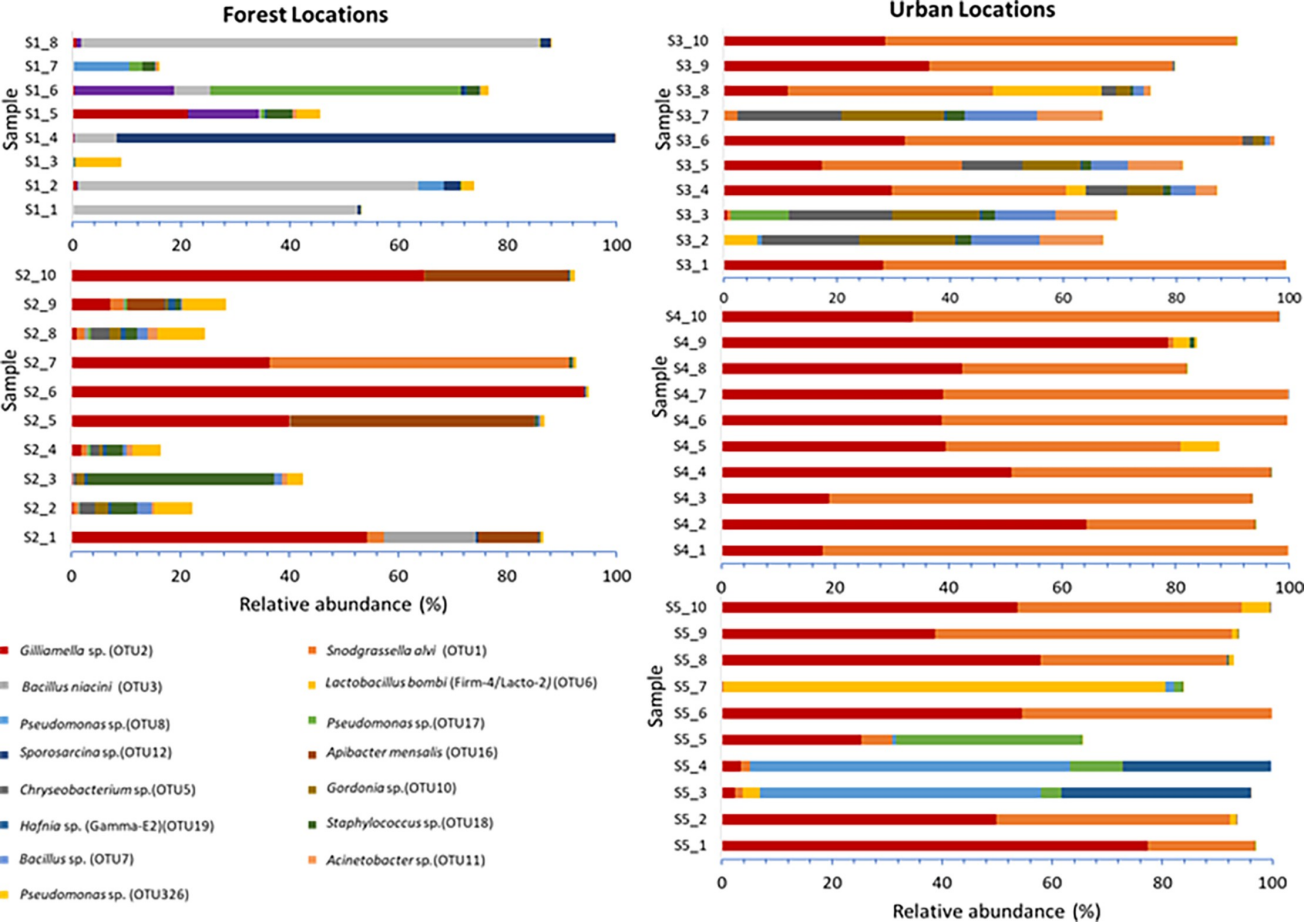
Gut bacterial community composition at the level of operational taxonomic units (OTUs) within the midgut and ileum in bumblebee queens (*Bombus terrestris*) from five different locations. Sampled locations represent two habitat types, including forest (S1-F and S2-F) and urbanized habitats (S3-U, S4-U and S5-U). Only the most abundant OTUs (i.e. with a mean sequence relative abundance > 1% over the entire dataset) are represented in the figure.

NMDS ordination of the gut bacterial community composition showed no clear separation among the different sampled areas (Bray-Curtis; stress = 0.16; Fig 2A). Indeed, also adonis analysis revealed no significant differences in bacterial community composition among queens from the different sites (*n* = 5; *R* = 0.259, *P* = 0.055). By contrast, significant differences were found between habitat types (*n* = 2; *R* = 0.957, *P* = 0.034). Gut bacterial communities in queens collected from the forest habitats showed a lower relative abundance of the core bacteria *Snodgrassella* (OTU1; Beta) (*P* = 0.041) and *Gilliamella* (OTU2; Gamma-1) (*P* = 0.011) (Fig 3), as was also confirmed by absolute quantifications using qPCR (data not shown). While being found in every specimen investigated, relative abundance of *Snodgrassella* and *Gilliamella* varied between 0.01 and 55.0% (mean: 5.4%) and between 0.03 and 94.1% (mean: 17.9%) for the forest specimens, while this was between 0.01 and 74.5% (mean: 35.9%) and between 0.03 and 78.7% (mean: 32.4%) for the urban specimens, respectively (Fig 3). When both taxa were taken together, mean relative abundance varied between 3.5% for the forest bees and 34.1% for the urban bee populations. Instead, forest specimens contained a higher relative amount of other bacteria, particularly members of *Bacilaceae*, *Flavobacteriaceae*, *Planococcaceae* and *Pseudomonaceae* (Fig 3; S3 Fig). For example, guts of forest specimens were particularly enriched with OTUs corresponding to *Bacillus niacini* (OTU3; *Bacilaceae*), *Cellvibrio* sp. (OTU53; *Pseudomonaceae*), *Flavobacterium* sp. (e.g. OTU30, 48 and 130; *Flavobacteriaceae*), *Pseudomonas* sp. (e.g. OTU 263, 326 and 1438; *Pseudomonaceae*), and *Sporosarcina* sp. (OTU12; *Planococcaceae*). Furthermore, an *Apibacter* OTU (*Apibacter mensalis*; OTU16; *Flavobacteriaceae*) represented a considerable fraction of the reads in some forest specimens (Fig 3; S3 Table).

Lactobacilli were found in 37 of the 48 investigated specimens (14 forest and 23 urban specimens). Nevertheless, despite their common occurrence, OTUs belonging to *Lactobacillaceae* occured at low relative abundance. Among these, the *Lactobacillus* phylotypes Firm-4/Lacto-2 (*Lactobacillus bombi*; OTU6; found in 21 specimens), Lacto-5 (*Lactobacillus* sp.; OTU20; found in 21 specimens) and Firm-5/Lacto-1 (*Lactobacillus bombicola*; OTU23; found in 16 specimens) were most prevalent (Table 2). Additionally, an OTU corresponding to *Lactobacillus iners* (OTU136) was frequently found (detected in 9 forest and 5 urban specimens). Further, a number of other lactobacilli were detected, albeit more eradically (Table 2). Likewise, bifidobacteria were sporadically detected, and were found to have low relative abundances (range: 0.001–2.0%). Specifically, we found bifidobacteria in 4 forest specimens (all from S1-F) and 11 urban specimens, with phylotype Bifido-3 (OTU24; *Bombiscardovia coagulans*) as the most prevalent OTU (present in 7 specimens) (Table 2). The phylogenetic position of the lactobacilli and bifidobacteria found in this study with regard to their closest family members is shown in S4 and S5 Figs, respectively. The Enterobacteriaceae core phylotypes Gamma-E1 (OTU9; *Buttiauxella* sp.) and Gamma-E2 (OTU19; *Hafnia* sp.) were commonly detected in our study. More particularly, the Gamma-E1 OTU was found in every specimen with the exception of two forest and two urban specimens. Phylotype Gamma-E2 was especially found in the forest specimens (17 out of 18 forest specimens *versus* 18 out of 30 urban specimens) (Table 2).

**Table 2:** Mean relative abundance and prevalence of previously described core bacteria^a^ in the midgut-ileum region of *Bombus terrestris* queens collected from five different locations, including two forest (S1-F and S2-F) and three urbanized habitats (S3-U, S4-U and S5-U).

OTU	Phylum	Family	Species^b^	Name OTU in literature^c^	Core in *B*. *terrestris*	S1-F		S2-F		S3-U		S4-U		S5-U	
						Rel. ab. (%)^d^	Present in host (*n* = 8)	Rel. ab. (%)	Present in host (*n* = 10)	Rel. ab. (%)	Present in host (*n* = 10)	Rel. ab. (%)	Present in host (*n* = 10)	Rel. ab. (%)	Present in host (*n* = 10)
24	Actinobacteria	*Bifidobacteriaceae*	*Bombiscardovia coagulans* (97.6%)	Bifido-3	Core	0.07	4	0.00	0	2.01	2	0.01	1	0.00	0
38			*Bifidobacterium bombi* (100%)	Bifido-2	Core	0.00	0	0.00	0	1.16	2	0.00	0	0.04	5
231			*Bifodobacterium animalis*, *B*. *thermacidophilum*, *B*. *thermophilum* (100%)			0.00	0	0.00	0	0.00	0	0.01	1	0.01	1
786			*Bifidobacterium actinocoloniiforme* (99.6%)	Bifido-1	Core	0.01	1	0.00	0	0.00	0	0.00	0	0.001	1
6	Firmicutes	*Lactobacillaceae*	*Lactobacillus bombi* (100%)	Firm-4/Lacto-2	Core	0.02	2	0.02	4	2.86	7	0.96	2	9.09	6
20			*Lactobacillus kimchicus*, *L*. *mixtipabuli*, *L*. *odoratitofui*, *L*. *pentosiphilus*, *L*. *silagei L*. *silagincola* (100%)	Lacto-5	Core	0.01	3	0.02	3	2.02	4	1.18	3	1.44	8
23			*Lactobacillus bombicola* (100%)	Firm-5/Lacto-1	Core	0.01	1	0.01	4	0.06	2	2.32	3	0.92	6
37			*Lactobacillus oligofermentans* (90%)	Lacto-4	Core	0.00	0	0.00	0	0.00	0	0.78	2	0.00	0
136			*Lactobacillus iners* (100%)			0.03	4	0.05	5	0.001	2	0.004	3	0.00	0
189			*Lactobacillus furfuricola*, *L*. *ginsenosidimutans*, *L*. *versmoldensis* (99.6%)			0.044	2	0.00	0	0.00	0	0.00	0	0.001	1
194			*Lactobacillus apis* (99.6%)			0.00	0	0.019	3	0.00	0	0.00	0	0.00	0
282			*Lactobacillus acidophilus*, *L*. *crispatus*, *L*. *gallinarum* (100%)			0.023	3	0.00	0	0.00	0	0.00	0	0.004	1
729			*Lactobacillus crustorum*, *L*. *farciminis*, *L*. *formosensis*, *L*. *heilongjiangensis*, *L*. *kimchiensis*, *L*. *mindensis*, *L*. *musae*, *L*. *nantensis*, *L*. *paralimentarius* (100%)			0.001	1	0.00	0	0.00	0	0.00	0	0.00	0
1304			*Lactobacillus delbrueckii* (100%)			0.005	1	0.001	1	0.00	0	0.00	0	0.001	2
1601			*Lactobacillus bombicola* (98%)			0.00	0	0.00	0	0.004	1	0.006	2	0.01	3
1697			*Lactobacillus fornicalis*, *L*. *jensenii* (99.6%)			0.009	1	0.00	0	0.00	0	0.00	0	0.00	0
9	Proteobacteria	Enterobacteriaceae	Several *Enterobacteriaceae* spp., including *Buttiauxella agrestis* (100%)	Gamma-E1	Core	0.34	6	1.095	10	1.69	10	0.23	8	0.23	10
19			Several *Enterobacteriaceae* spp., including *Hafnia (100%)*	Gamma-E2	Core	0.18	7	0.44	10	0.19	8	0.01	3	6.14	7
1		Neisseriaceae	*Snodgrassella alvi* (98.8%)	Beta	Core	4.10	8	6.40	10	33.10	10	50.01	10	24.43	10
2		Orbaceae	*Gilliamella apicola*, *G*. *bombi*, *G*. *bombicola; G*. *mensalis* (100%)	Gamma-1	Core	2.91	8	29.97	10	18.38	10	42.39	10	36.32	10

a According to Cariveau et al. [26] and Meeus et al. [27]. Additionally, all other Lactobacillaceae en Bifidobacteriaceae OTUs found in this study are reported.

b Nearest neighbor based on a BLAST search in GenBank against type strains. Percentage of sequence identity (on a total of 250 bp) is reported between brackets.

c As used by Meeus et al. [27].

d Mean relative abundance, calculated based on specimens containing the respective OTU.

### Fungal community composition

Whereas bacterial OTUs were found in every specimen, fungal taxa (both true fungi and yeasts) were only detected in *B*. *terrestris* queens from the forest locations S1-F and S2-F (fungi present in every specimen analyzed), and in two individuals from site S3–U (S1 Table). Illumina sequencing revealed a total of 107 fungal OTUs belonging to three phyla, including Ascomycota, Basidiomycota and Zygomycota. No differences were observed in OTU richness, evenness and community structure between bees from both forest locations (*P* = 0.063) (Fig 2B; S6 Fig), and a mean fungal richness of 15 OTUs per specimen was recorded (range between 6 and 25 OTUs) (S1 Table). The most prevalent fungi and yeasts detected included widespread taxa such as *Saccharomyces* (Ascomycota; *Saccharomycetaceae*), *Trichoderma* (Ascomycota; *Hypocreaceae*) and *Mucor* (Zygomycota; *Mucoraceae*) (S4 Table).

### Pathogen assessment and association with habitat type and specific bacteria

**Fig 4:**
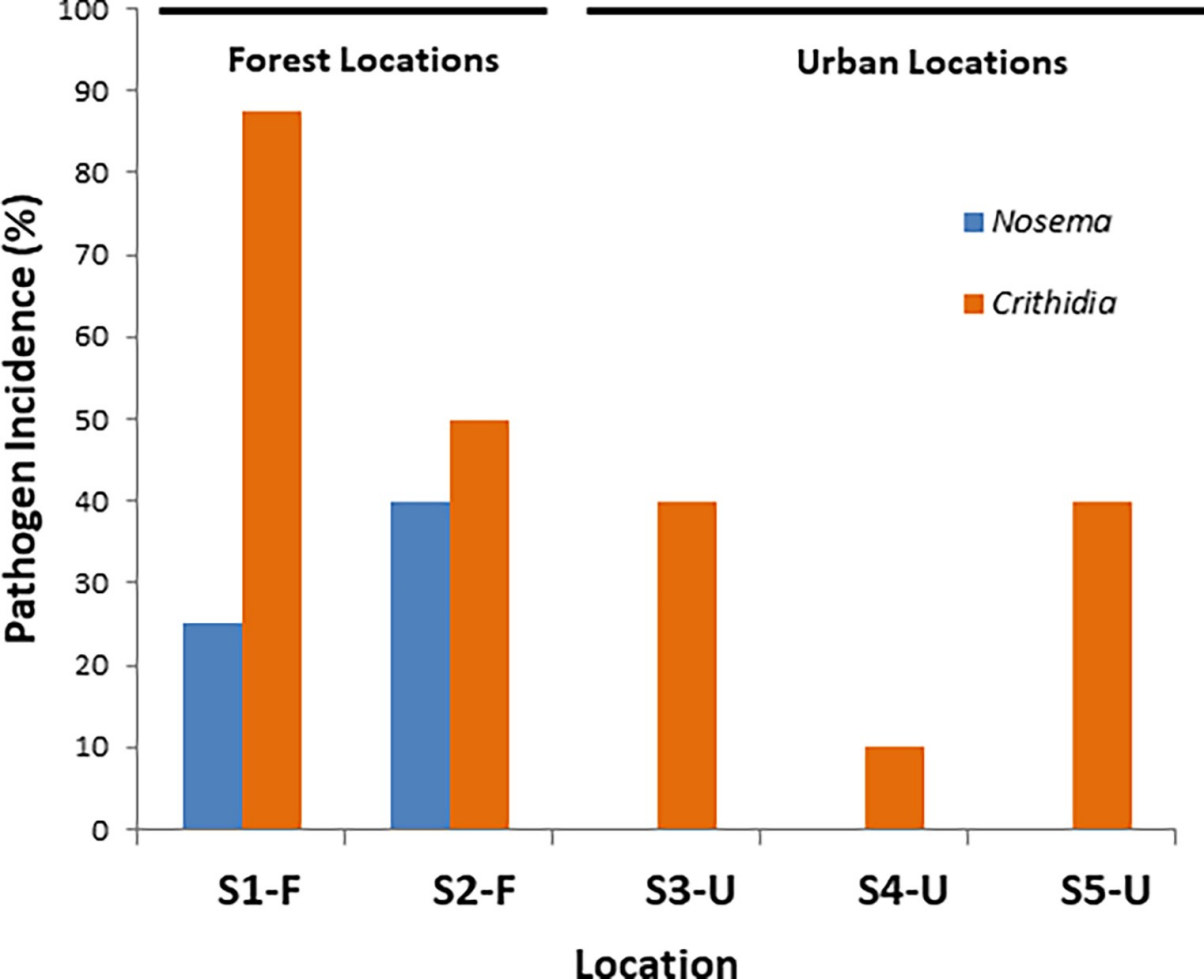
Pathogen incidence (%) in bumblebee queens (*Bombus terrestris*) from five different locations. Sampled locations represent two habitat types, including forest (S1-F and S2-F) and urbanized habitats (S3-U, S4-U and S5-U).

**Fig 5:**
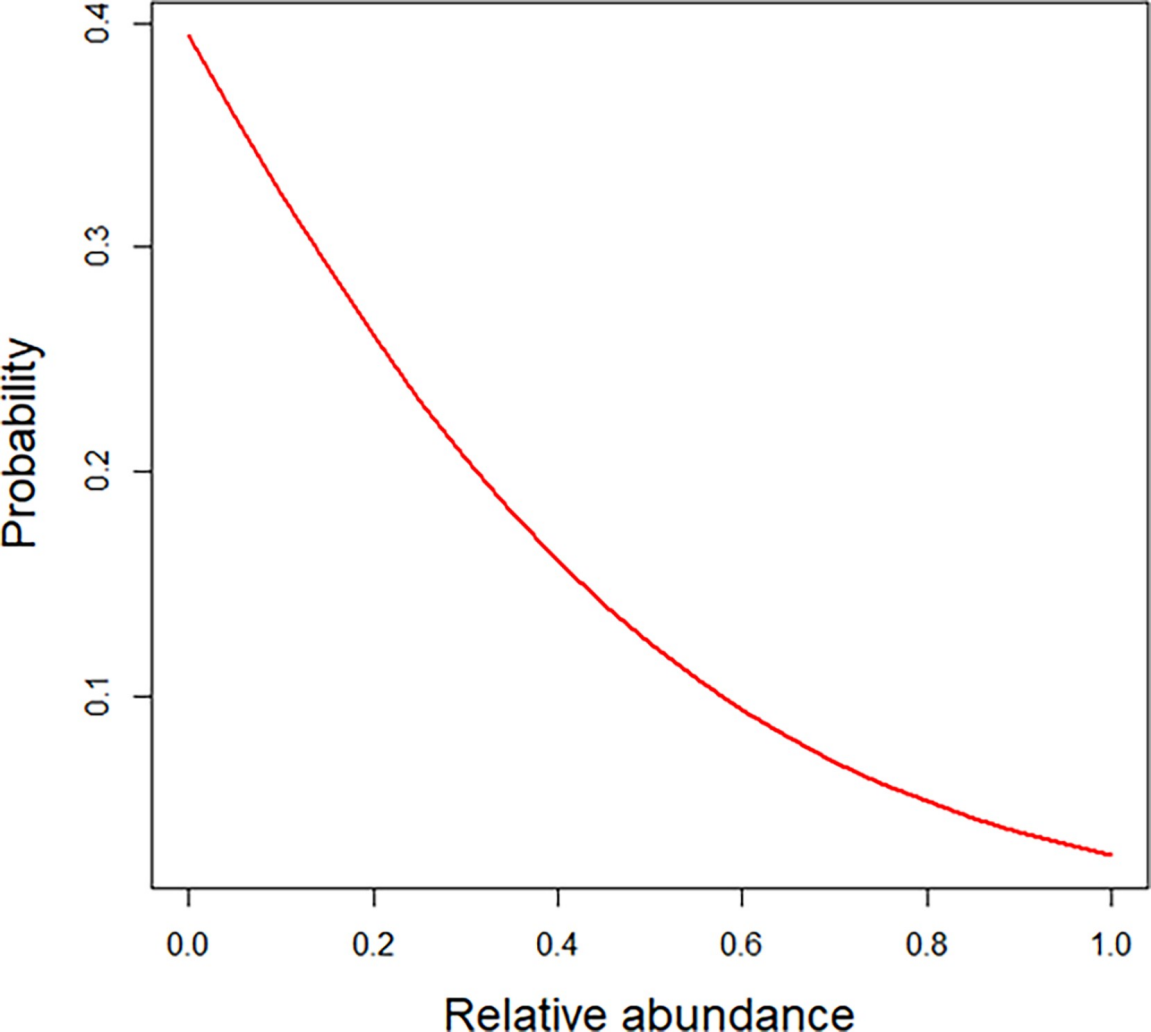
Probability of infection by *Nosema bombi* in function of the relative abundance of *Snodgrassella*. Presence of *Nosema* is negatively and significantly (*P* < 0.001) related to the relative abundance of *Snodgrassella*.

Pathogen screening using qPCR revealed the presence of *N*. *bombi* and *C*. *bombi* in 4 (8.3%) and 20 (43.8%) of the 48 investigated queens, repectively. Two specimens were found to contain both pathogens (S1 Table). *Nosema* was only found in forest specimens. Further, incidence of *Crithidia* was significantly higher (*P* = 0.03) in specimens from the forest locations than from urbanized locations (Fig 4). The occurrence of both pathogens in the gut of bumblebees was not significantly (*P* > 0.05) related to bacterial diversity in the gut. On the other hand, presence of *Nosema* was negatively and significantly (*P* < 0.001) related to the relative abundance of *Snodgrassella* (OTU1) (Fig 5).

## Discussion

Knowledge about the health of bees and bumblebees is important as they are among the most important pollinators for many native plants as well as several crops [42, 43]. Wild bee and bumblebee populations have been in a steady decline worldwide [7], and among several factors, land-use change and increased pathogen prevalence have been proposed to contribute to bee decline [8, 9, 44]. As anthropogenic use of land is likely to increase, we investigated the impact of urbanization on the gut microbial community composition of wild *B*. *terrestris* queens, and investigated whether relationships could be established with pathogen infection.

### Core bacteria

Gut microbial community analysis revealed several bacterial taxa known to be associated with bees and bumblebees. *Snodgrassella*, *Gilliamella*, *Lactobacillus* Firm-4/Lacto-2 and Firm-5/Lacto-1 and *Bifidobacterium* have been described as core bacteria of *Apis* and *Bombus* hosts [26, 27, 45, 46]. The former two were also highly prevalent in the *B*. *terrestris* queens investigated in this study, confirming their strong association with (bumble)bees [27, 47]. These microbes are vertically transmitted from a mother colony to the offspring and have not been detected outside of bees [48]. Additionally, the bee gut symbiotic lactobacilli Firm-4/Lacto-2, Firm-5/Lacto-1 and Lacto-5 were commonly detected next to a number of environmental lactobacilli with a more erratic occurrence. Recently, Bifidobacteria were proposed as core bacteria in *B*. *terrestris* [27]. However, our data does not seem to support this scenario. Bifidobacteria occurred at low relative abundance, and were only detected in 15 out of the 48 investigated queens. Most probably, the low prevalence and low relative abundance of bifidobacteria can be explained by the fact that we focused on midgut and ileum, while other studies generally investigated the microbiome of whole guts, including rectum. Previous research has shown that the midgut of social bees only contains few bacteria, while the ileum and rectum are strongly colonized by bacteria, totaling up to 10^8^ and 10^9^ bacterial cells, respectively [49]. Furthermore, while the ileum is dominated by *Snodgrassella*, *Gilliamella*, and the lactobacilli Firm-4/Lacto-2 and Firm-5/Lacto-1, the rectum is dominated by lactobacilli and bifidobacteria [49]. Therefore, as the rectum was not taken into account in our study, this may explain the low prevalence and abundance of bifidobacteria. Additionally, it cannot be excluded that age differences contributed to this discrepancy [50], but further research is needed to confirm this. By contrast, the *Enterobacteriaceae* OTUs corresponding to Gamma-E1 and Gamma-E2 had a high prevalence (present in 44 and 35 specimens, respectively), corroborating previous results [27].

### Impact of habitat type

The gut microbial community composition from *B*. *terrestris* queens differed strongly between bumblebees from forests and urbanized habitats. First, fungi were almost exclusively associated with bumblebee queens from forests. Within these fungal communities, *Saccharomyces* yeasts were dominant (mean average relative abundance of 17.3%) and occurred in every specimen investigated. In previous studies, insect-associated yeasts such as *Saccharomyces* were reported to play an important role in food digestion and detoxification of toxic plant metabolites in the insect host [51]. Further, environmental fungi like *Trichoderma* that are commonly encountered in forest soils and airborne fungi like *Mucor* and *Penicillium* were found. So far it is not clear why fungi were strongly associated with forest specimens, but lower bacterial concentrations in these samples may (partially) explain this observation (less competition). Secondly, there was a striking difference in gut bacterial community composition. Bacterial richness was highest for the bumblebee queens occurring in the forest locations (on average 239 OTUs per specimen); queens from the urbanized areas contained *c*. 75% less bacterial OTUs (on average 56 OTUs per specimen). Gut communities of the latter were predominantly composed of the core bacteria *Snodgrassella* and *Gilliamella*, representing a combined relative abundance up to 68.2% per specimen. Forest specimens had a lower relative abundance of both bacteria, as were absolute levels. Instead, forest specimens harboured a huge diversity of non-core, environmental bacteria. Recent research has shown that flowers represent an important hot spot of transmission of environmental microbes to bee guts [22], and it is reasonable to assume that the likelihood of transmitting novel microbes to the bee gut is higher in natural environments. Natural habitats like forest environments are generally characterized by dense patches of flowers that are frequently visited and shared by diverse insects [52], facilitating transmission of new and additional microbes to the gut microbiome, e.g. by ingestion of microbe-contaminated nectar or pollen [23, 53, 54]. Guts of forest specimens were enriched with opportunistic bacteria like pseudomonads and *Flavobacteriacae* of which several members have been found in flowers [54–56]. Several *Pseudomonaceae* and *Flavobacteriaceae* OTUs showed high 16S rRNA gene sequence identity (up to 100%) with strains that have been found in nectar or on pollen (data not shown). At the time of sampling, however, there were not many plant species flowering, and flowers were not investigated for microbial presence. Further investigations are needed to explain the differences in microbial gut community composition between both types of habitat.

So far, only very little is known about the functional potential of these environmental microbes in the bee gut. However, in a recent study it was found that several of such microorgansms may have antimicrobial activity and can help protect against microbial pathogens and parasites [17]. Further research is needed to unravel the possible functions of these bacteria, and to find out whether or not they impact on bee fitness. However, it has to be noted that our data do not imply that all opportunistic taxa found are able to replicate and to stably colonize the gut. Possibly they are dead microbes consumed with pollen or nectar, or microorganisms that became inactivated in the harsh environment of the bee gut. Further, it is unknown whether the investigated bumblebee specimens had the same age or experienced different levels of stress, two factors that may affect gut communities [57].

### Association with pathogen infection

Gut microbiota are critical for the health of many insect species [58]. In this regard, overall microbiota richness has been assumed to be important in microbial gut function. For example, in locusts it has been demonstrated that increasing diversity of gut bacteria reduces susceptibility to a pathogen [59]. Likewise, using experimental transplantations in *Bombus impatiens*, Mockler *et al*. [30] showed that lower *Crithidia* infection loads were associated with high microbiome diversity and large gut bacterial populations. Similar results were obtained by Praet *et al*. [17] although bacterial gut isolates from wild bumblebees were screened *in vitro* in pectin degradation assays and pathogen growth inhibition assays. By contrast, Koch *et al*. [48] found in their *in vivo* assays that microbiota richness was positively associated with *Crithidia* infection in *B*. *terrestris*. Our results, however, did not show a significant relationship between pathogen infection and microbial diversity. On the other hand, we found a significant and negative relationship between *Nosema* prevalence and relative abundance of the core resident *Snodgrassella*. This is in line with previous research that has shown *Snodgrassella* to be negatively associated with *N*. *bombi* in *B*. *terrestris* [60]. Potentially, this can be explained by the fact that *Snodgrassella* (as well as *Gilliamella*) is able to form biofilm-layers on the host epithelium of the gut, by which pathogen infection may be restricted [25]. In contrast to other studies [24, 26, 30, 61], no relationship was found between the prevalence of cre gut residents and *Crithidia* infection.

## Conclusion

Overall, we observed a striking difference between the gut communities within the midgut and ileum of *B*. *terrestris* queens living in urban environments compared to those in forest environments, both for fungi and bacteria. Furthermore, our study provides evidence that the core resident *Snodgrassella* may have a protective effect against pathogens, and suggests that pathogens may be more prevalent in specimens from natural environments, which may be contradictory to the general assumption of a forest as a source of “pristine”, healthy specimens. Further research with more locations, however, is needed to draw strong conclusions regarding the effect of urbanization on the structure of bumblebee gut commities and bumblebee health and fitness. Possible factors include: landscape structure, pesticide exposure, quantity/ quality and connectivity of food sources, exposure to other arthropod fauna, and other symbiont or pathogen transmission routes. Furthermore, it remains to be investigated whether the same trends will be observed when the rectum is taken into account. Also, additional research using microbial isolates is needed to unravel the precise function of the environmental opportunistic microbes found, and to assess their role in bee health and overall bee fitness.

## Supporting information

S1 Table Overview of samples (*Bombus terrestris* queens) investigated in this study, including sampling information and diversity measures. (XLSX)

S2 Table Primer design and sample-specific barcodes. (XLSX)

S3 Table Identification of bacterial operational taxonomic units (OTUs) according to the Silva v1.23 database and distribution over the investigated samples. (XLSX)

S4 Table Identification of fungal operational taxonomic units (OTUs) according to the RDP Warcup fungal ITS training set (v2) and distribution over the investigated samples. (XLSX)

S1 Fig Relative abundance (%) of the bacterial phyla found in the guts (midgut and ileum) of bumblebee queens (*Bombus terrestris*) from five different locations. Sampled locations represent two habitat types, including forest (S1-F and S2-F) and urbanized habitats (S3-U, S4-U and S5-U).
(TIF)

S2 Fig **Rarefaction curves showing the number of gut bacterial (A) and fungal (B) operational taxonomic units (OTUs) per bumblebee queen (*Bombus terrestris*) from five different locations**. Sampled locations represent two habitat types, including forest (S1-F (yellow) and S2-F (orange)) and urbanized habitats (S3-U (purple), S4-U (dark blue) and S5-U (light blue)). Rarefaction curves reached saturation, suggesting that the most abundant community members were covered by our sequencing depth.
(TIF)

S3 Fig Relative abundance (%) of bacterial families in the guts (midgut and ileum) of bumblebee queens (*Bombus terrestris*) from five different locations. Sampled locations represent two habitat types, including forest (S1-F and S2-F) and urbanized habitats (S3-U, S4-U and S5-U).
(TIF)

S4 Fig Maximum-likelihood tree derived from the V4 region of the 16S rRNA gene sequence (250 bp) of *Lactobacillaceae* occurring in the digestive tract of *Bombus* and *Apis*, showing the phylogenetic position of the lactobacilli found in this study (OTU6, 20, 23, 37, 136, 189, 194, 282, 729, 1304, 1601 and 1697). Type strains of the closest relatives were also included in the tree.
(PDF)

S5 Fig Maximum-likelihood tree derived from the V4 region of the 16S rRNA gene sequence (250 bp) of *Bifidobacteriaceae* occurring in the digestive tract of *Bombus*, showing the phylogenetic position of the bifidobacteria found in this study (OTU24, 38 and 786). Type strains of the closest relatives were also included in the tree.
(PDF)

S6 Fig Fungal community composition at the level of operational taxonomic units (OTUs) within the midgut and ileum in bumblebee queens (*Bombus terrestris*) having fungi. Specimens with fungi were caught in the forest habitats S1-F and S2-F (18 specimens) and the urbanized habitat S3-U (2 specimens). All other investigated bumblebees were negative for fungi. Only the most abundant OTUs (i.e. with a mean sequence relative abundance > 1% over the entire dataset) are represented in the figure.
(PPTX)
