# Supplementary figures and images for: Habitat-specific variation in gut microbial communities and pathogen prevalence in bumblebee queens (Bombus terrestris)
Source: PLoS One. 2018 Oct 25;13(10):e0204612. doi: 10.1371/journal.pone.0204612 (PMC6201867; doi:10.1371/journal.pone.0204612)

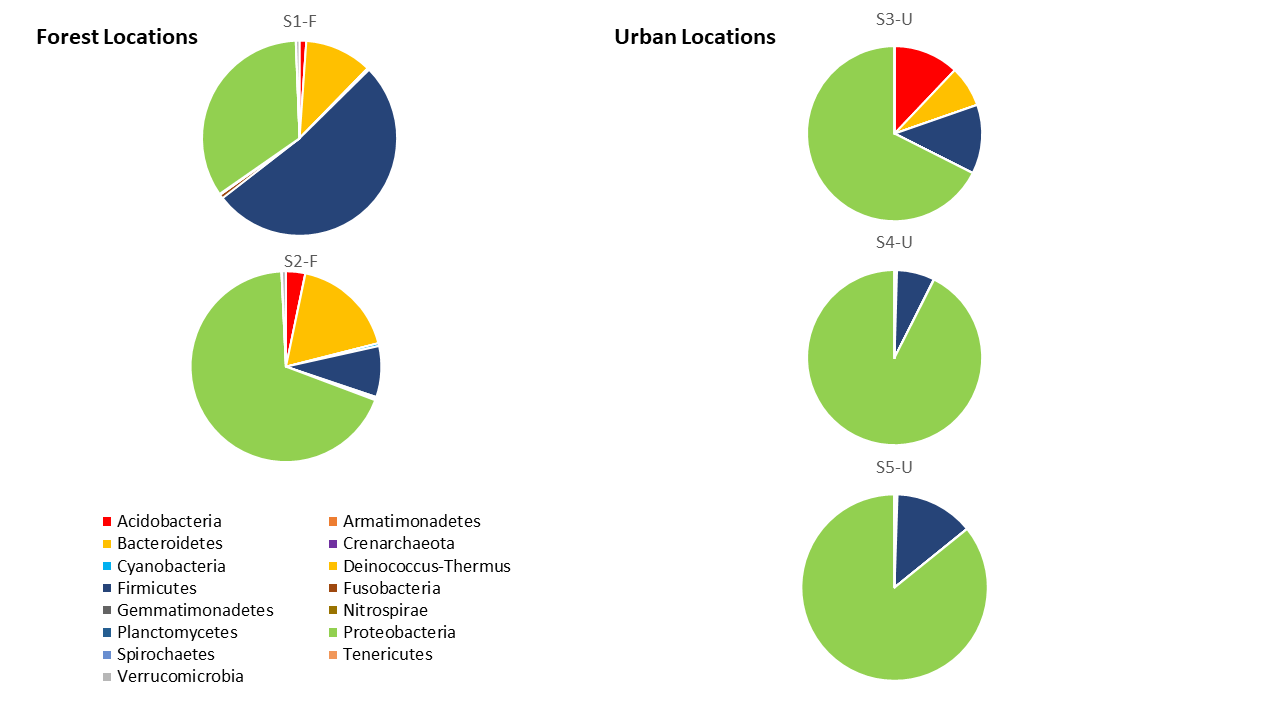

Supplement: S1 Fig — Sampled locations represent two habitat types, including forest (S1-F and S2-F) and urbanized habitats (S3-U, S4-U and S5-U). (TIF) [file pone.0204612.s005.tif]

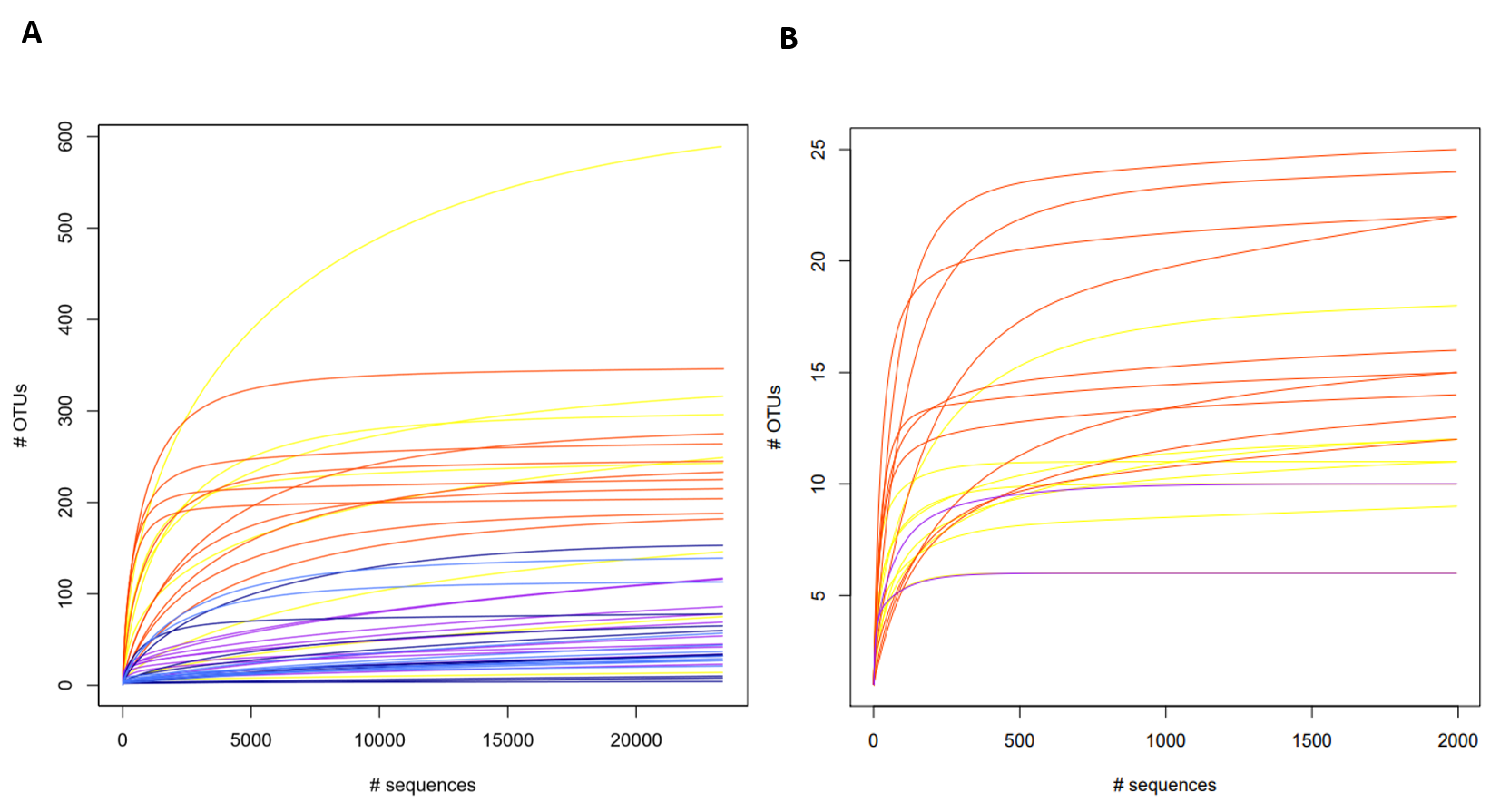

Supplement: S2 Fig — Rarefaction curves showing the number of gut bacterial (A) and fungal (B) operational taxonomic units (OTUs) per bumblebee queen (Bombus terrestris) from five different locations. Sampled locations represent two habitat types, including forest (S1-F (yellow) and S2-F (orange)) and urbanized habitats (S3-U (purple), S4-U (dark blue) and S5-U (light blue)). Rarefaction curves reached saturation, suggesting that the most abundant community members were covered by our sequencing depth. (TIF) [file pone.0204612.s006.tif]

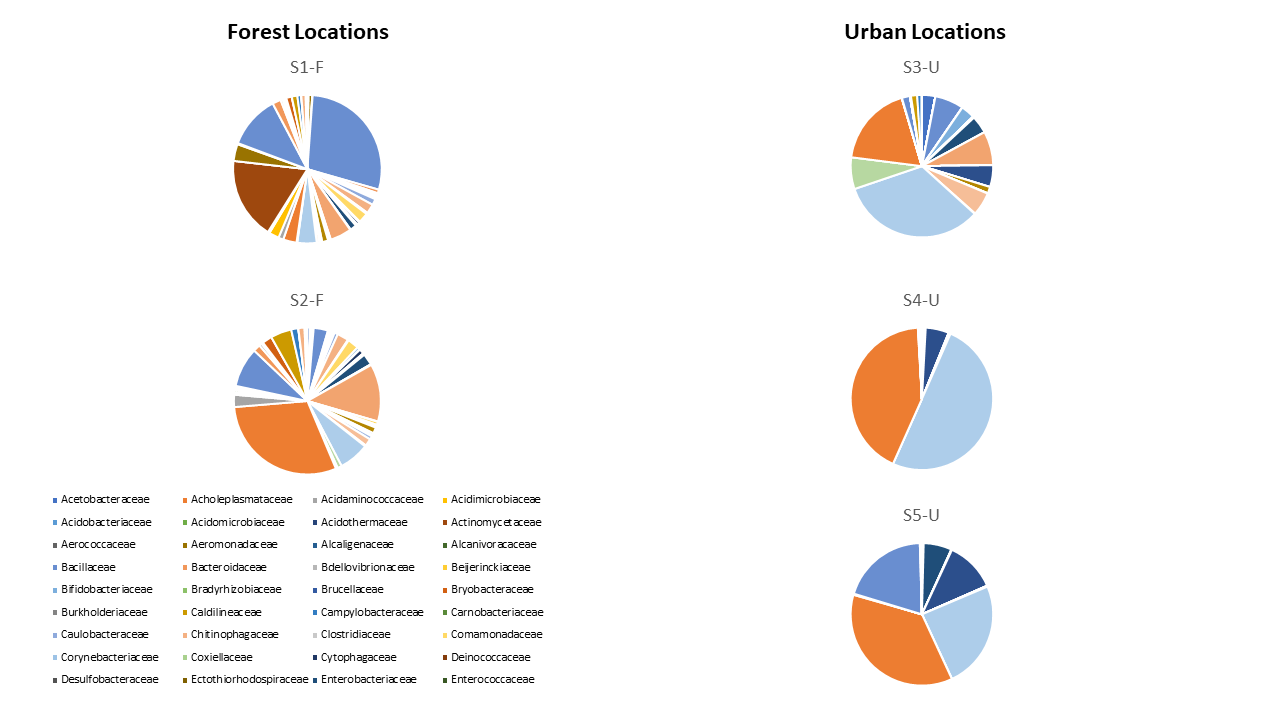

Supplement: S3 Fig — Sampled locations represent two habitat types, including forest (S1-F and S2-F) and urbanized habitats (S3-U, S4-U and S5-U). (TIF) [file pone.0204612.s007.tif]

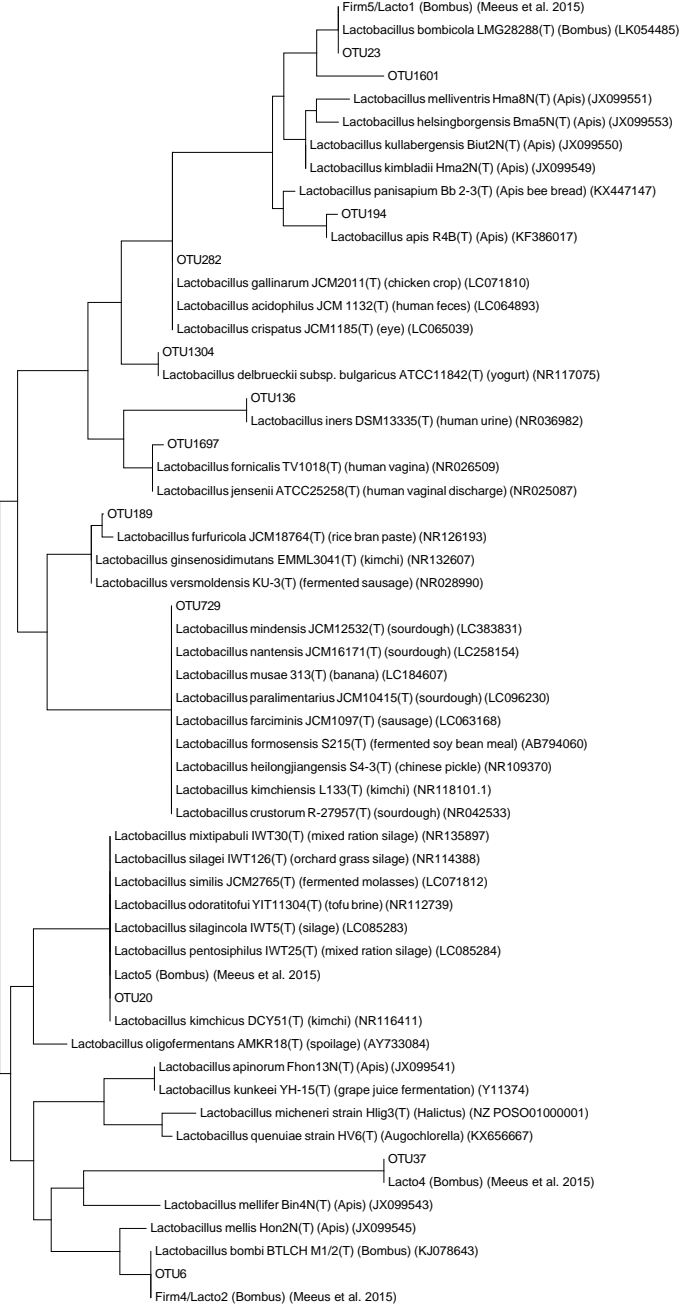

0.0100

Supplement: S4 Fig — Type strains of the closest relatives were also included in the tree. (PDF) [file pone.0204612.s008.pdf]

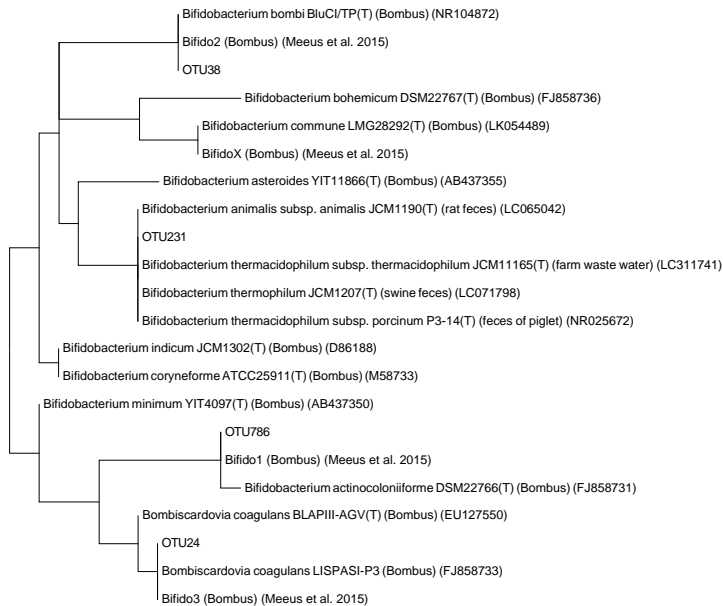

0.0050

Supplement: S5 Fig — Type strains of the closest relatives were also included in the tree. (PDF) [file pone.0204612.s009.pdf]
