# Supplementary material for: Habitat-specific variation in gut microbial communities and pathogen prevalence in bumblebee queens (Bombus terrestris)
Source: PLoS One. 2018 Oct 25;13(10):e0204612. doi: 10.1371/journal.pone.0204612 (PMC6201867; doi:10.1371/journal.pone.0204612)
Supplement: S6 Fig — Specimens with fungi were caught in the forest habitats S1-F and S2-F (18 specimens) and the urbanized habitat S3-U (2 specimens). All other investigated bumblebees were negative for fungi. Only the most abundant OTUs (i.e. with a mean sequence relative abundance > 1% over the entire dataset) are represented in the figure. (PPTX) [file pone.0204612.s010.pptx]

## Slide 1
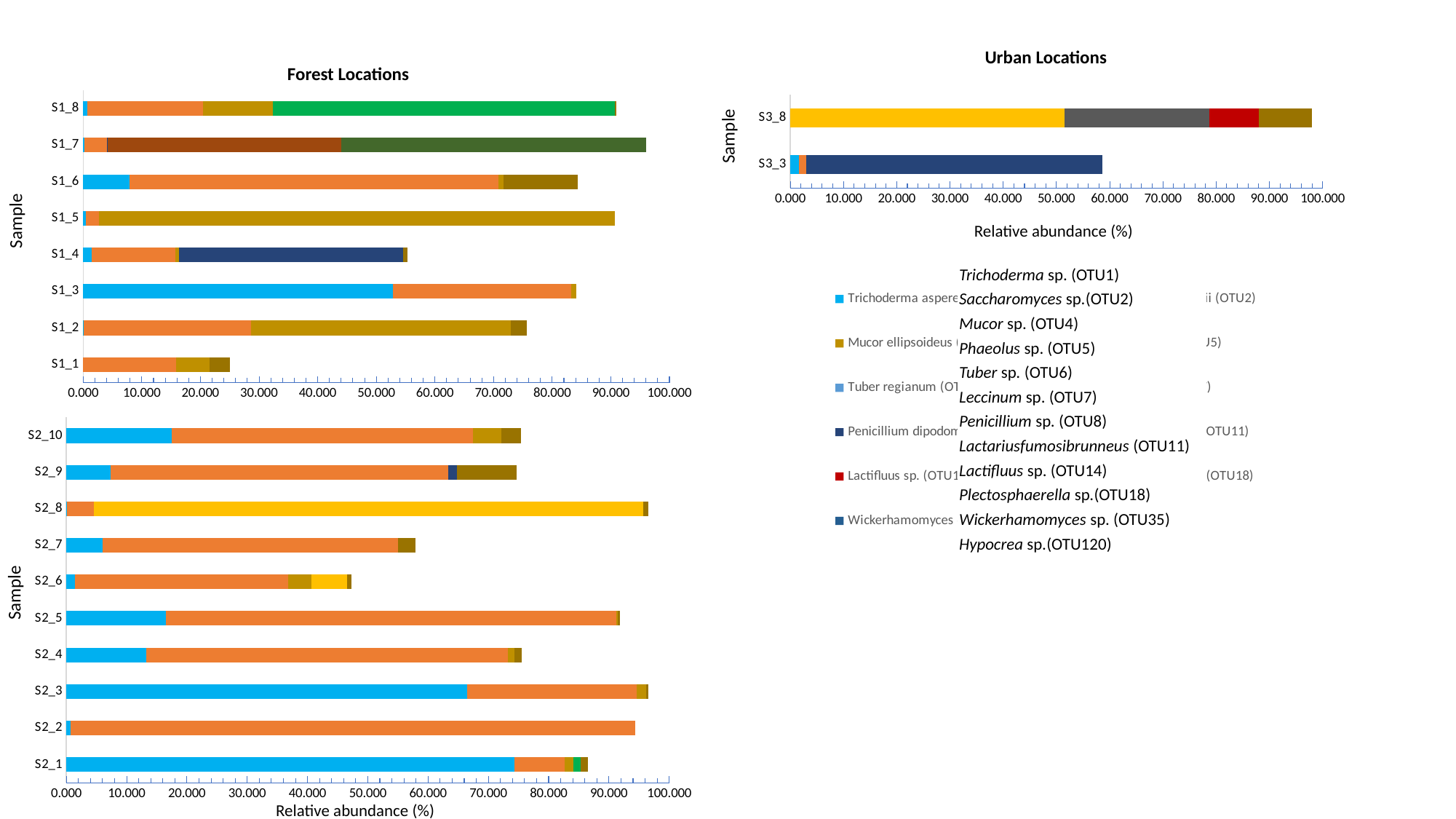

Urban Locations
### Chart
| Category | Trichoderma asperelloides (OTU1) | Saccharomyces kudriavzevii (OTU2) | Mucor ellipsoideus (OTU4) | Phaeolus schweinitzii (OTU5) | Tuber regianum (OTU6) | Leccinum rndifoliae (OTU7) | Penicillium dipodomyis (OTU8) | Lactariusfumosibrunneus (OTU11) | Lactifluus sp. (OTU14) | Plectosphaerella plurivora (OTU18) | Wickerhamomyces ciferrii (OTU35) | Hypocrea lixii (OTU120) |
|---|---|---|---|---|---|---|---|---|---|---|---|---|
| S1_1 | 0.0 | 15.897693079237712 | 5.616850551654965 | 0.0 | 0.0 | 0.0 | 0.0 | 0.0 | 0.0 | 3.460381143430291 | 0.0 | 0.0 |
| S1_2 | 0.05015045135406219 | 28.635907723169506 | 44.28284854563691 | 0.0 | 0.0 | 0.0 | 0.0 | 0.0 | 0.0 | 2.7582748244734203 | 0.0 | 0.0 |
| S1_3 | 52.80842527582749 | 30.391173520561686 | 0.9528585757271816 | 0.0 | 0.0 | 0.0 | 0.0 | 0.0 | 0.0 | 0.0 | 0.0 | 0.0 |
| S1_4 | 1.5045135406218655 | 14.24272818455366 | 0.6018054162487462 | 0.0 | 0.0 | 0.0 | 38.214643931795386 | 0.0 | 0.0 | 0.802407221664995 | 0.0 | 0.0 |
| S1_5 | 0.4012036108324975 | 2.3069207622868606 | 87.96389167502508 | 0.0 | 0.0 | 0.0 | 0.0 | 0.0 | 0.0 | 0.0 | 0.0 | 0.0 |
| S1_6 | 7.923771313941826 | 62.938816449348046 | 0.8525576730190572 | 0.0 | 0.0 | 0.0 | 0.0 | 0.0 | 0.0 | 12.688064192577734 | 0.0 | 0.0 |
| S1_7 | 0.15045135406218654 | 3.911735205616851 | 0.0 | 0.0 | 0.0 | 0.0 | 0.15045135406218654 | 39.81945837512538 | 0.0 | 0.0 | 0.0 | 51.955867602808425 |
| S1_8 | 0.7522567703109327 | 19.709127382146438 | 11.885656970912738 | 0.0 | 0.0 | 58.37512537612839 | 0.0 | 0.0 | 0.0 | 0.25075225677031093 | 0.0 | 0.0 |Forest Locations
### Chart
| Category | Trichoderma asperelloides (OTU1) | Saccharomyces kudriavzevii (OTU2) | Mucor ellipsoideus (OTU4) | Phaeolus schweinitzii (OTU5) | Tuber regianum (OTU6) | Leccinum rndifoliae (OTU7) | Penicillium dipodomyis (OTU8) | Lactariusfumosibrunneus (OTU11) | Lactifluus sp. (OTU14) | Plectosphaerella plurivora (OTU18) | Wickerhamomyces ciferrii (OTU35) | Hypocrea lixii (OTU120) |
|---|---|---|---|---|---|---|---|---|---|---|---|---|
| S3_3 | 1.6549648946840523 | 1.354062186559679 | 0.0 | 0.0 | 0.0 | 0.0 | 55.61685055165496 | 0.0 | 0.0 | 0.0 | 0.0 | 0.0 |
| S3_8 | 0.0 | 0.0 | 0.0 | 51.504513540621865 | 0.0 | 0.0 | 0.0 | 27.181544633901705 | 9.327983951855568 | 9.979939819458375 | 0.0 | 0.0 |Sample
Sample
Relative abundance (%)
| Trichoderma sp. (OTU1) |
| --- |
| Saccharomyces sp.(OTU2) |
| Mucor sp. (OTU4) |
| Phaeolus sp. (OTU5) |
| Tuber sp. (OTU6) |
| Leccinum sp. (OTU7) |
| Penicillium sp. (OTU8) |
| Lactariusfumosibrunneus (OTU11) |
| Lactifluus sp. (OTU14) |
| Plectosphaerella sp.(OTU18) |
| Wickerhamomyces sp. (OTU35) |
| Hypocrea sp.(OTU120) |
### Chart
| Category | Trichoderma asperelloides (OTU1) | Saccharomyces kudriavzevii (OTU2) | Mucor ellipsoideus (OTU4) | Phaeolus schweinitzii (OTU5) | Tuber regianum (OTU6) | Leccinum rndifoliae (OTU7) | Penicillium dipodomyis (OTU8) | Lactariusfumosibrunneus (OTU11) | Lactifluus sp. (OTU14) | Plectosphaerella plurivora (OTU18) | Wickerhamomyces ciferrii (OTU35) | Hypocrea lixii (OTU120) |
|---|---|---|---|---|---|---|---|---|---|---|---|---|
| S2_1 | 74.37311935807422 | 8.224674022066198 | 1.5546639919759278 | 0.0 | 0.0 | 1.2036108324974923 | 0.0 | 0.0 | 0.0 | 1.1534603811434303 | 0.0 | 0.0 |
| S2_2 | 0.7522567703109327 | 93.58074222668003 | 0.0 | 0.0 | 0.0 | 0.0 | 0.0 | 0.0 | 0.0 | 0.0 | 0.0 | 0.0 |
| S2_3 | 66.49949849548646 | 28.13440320962889 | 1.5546639919759278 | 0.0 | 0.0 | 0.0 | 0.0 | 0.0 | 0.0 | 0.4012036108324975 | 0.0 | 0.0 |
| S2_4 | 13.28986960882648 | 59.929789368104316 | 1.1534603811434303 | 0.0 | 0.0 | 0.0 | 0.0 | 0.0 | 0.0 | 1.2036108324974923 | 0.0 | 0.0 |
| S2_5 | 16.49949849548646 | 74.6740220661986 | 0.25075225677031093 | 0.0 | 0.0 | 0.0 | 0.0 | 0.0 | 0.0 | 0.4513540621865597 | 0.0 | 0.0 |
| S2_6 | 1.4543630892678034 | 35.30591775325978 | 3.911735205616851 | 5.917753259779338 | 0.0 | 0.0 | 0.0 | 0.0 | 0.0 | 0.7522567703109327 | 0.0 | 0.0 |
| S2_7 | 6.018054162487462 | 49.047141424272816 | 0.0 | 0.0 | 0.0 | 0.0 | 0.0 | 0.0 | 0.0 | 2.8585757271815444 | 0.0 | 0.0 |
| S2_8 | 0.10030090270812438 | 4.513540621865597 | 0.0 | 91.02306920762287 | 0.0 | 0.0 | 0.10030090270812438 | 0.0 | 0.0 | 0.7522567703109327 | 0.0 | 0.0 |
| S2_9 | 7.3219658976930795 | 55.9679037111334 | 0.0 | 0.0 | 0.0 | 0.0 | 1.5045135406218655 | 0.0 | 0.0 | 9.87963891675025 | 0.0 | 0.0 |
| S2_10 | 17.45235707121364 | 50.0 | 4.714142427281845 | 0.0 | 0.0 | 0.0 | 0.0 | 0.0 | 0.0 | 3.20962888665998 | 0.0 | 0.0 |Sample
Relative abundance (%)
